# Development and validation of a prediction model of perioperative hypoglycemia risk in patients with type 2 diabetes undergoing elective surgery

**DOI:** 10.1186/s12893-022-01601-3

**Published:** 2022-05-10

**Authors:** Huiwu Han, Juan Lai, Cheng Yan, Xing Li, Shuoting Hu, Yan He, Hong Li

**Affiliations:** 1 Teaching and Research Section of Clinical Nursing, Xiangya Hospital of Central South University, Changsha, Hunan People’s Republic of China; 2 National Clinical Research Center for Geriatric Disorders, Xiangya Hospital, Central South University, Changsha, Hunan People’s Republic of China; 3 Institute of Hospital Management, Central South University, Changsha, Hunan People’s Republic of China; 4 Cardiovascular Medicine Department, Xiangya Hospital at Central South University, Changsha, Hunan People’s Republic of China; 5 Nursing Department, The People’s Hospital of Liuyang, Hunan, People’s Republic of China

**Keywords:** Type 2 diabetes mellitus (T2DM), Elective surgery, Perioperative period, Hypoglycemia, Risk prediction model

## Abstract

**Aim:**

To develop and validate a prediction model to evaluate the perioperative hypoglycemia risk in hospitalized type 2 diabetes mellitus (T2DM) patients undergoing elective surgery.

**Methods:**

We retrospectively analyzed the electronic medical records of 1410 T2DM patients who had been hospitalized and undergone elective surgery. Regression analysis was used to develop a predictive model for perioperative hypoglycemia risk. The receiver operating characteristic (ROC) curve and the Hosmer–Lemeshow test were used to verify the model.

**Results:**

Our study showed an incidence of 10.7% for level 1 hypoglycemia and 1.8% for level 2 severe hypoglycemia during the perioperative period. A perioperative hypoglycemic risk prediction model was developed that was mainly composed of four predictors: duration of diabetes ≥ 10 year, body mass index (BMI) < 18.5 kg/m^2^, standard deviation of blood glucose (SDBG) ≥ 3.0 mmol/L, and preoperative hypoglycemic regimen of insulin subcutaneous. Based on this model, patients were categorized into three groups: low, medium, and high risk. Internal validation of the prediction model showed high discrimination (ROC statistic = 0.715) and good calibration (no significant differences between predicted and observed risk: Pearson χ^2^ goodness-of-fit P = 0.765).

**Conclusions:**

The perioperative hypoglycemic risk prediction model categorizes the risk of hypoglycemia using only four predictors and shows good reliability and validity. The model serves as a favorable tool for clinicians to predict hypoglycemic risk and guide future interventions to reduce hypoglycemia risk.

## Introduction

Diabetes mellitus is a growing global health problem with high prevalence, high disability, high mortality, and high disease burden, and has been listed as the ninth major cause of death [1, 2]. According to the International Diabetes Federation (IDF) estimates [3], 1 in 11 adults, or 415 million adults in the world had diabetes mellitus in 2015, which is projected to rise to 642 million by 2040. Type 2 diabetes mellitus (T2DM) accounted for over 90% of the total diabetes mellitus cases and contributed significantly to the global disease burden [1, 4]. China has been the top epicenter for the global epidemic of T2DM, with a prevalence of 11.6% among the adult population based on a recent large-scale population-based survey [5]. The prevalence of T2DM is even higher among surgical patients, reaching as high as 25% [6], and is associated with 5–6 times increased risk of perioperative complications and mortality as compared to non-diabetic patients [7].

Hypoglycemia is one of the most prevalent perioperative complications in patients with type 2 diabetes (T2DM), with an incidence ranging from 5.1 to 25.3% [8]. Hypoglycemia in the perioperative period is associated with a wide range of adverse outcomes including cognitive dysfunction, brain damage, cardiovascular events, morbidity, and even death [5, 9, 10]. For instance, when blood glucose decreases to 70 mg/dL (3.9 mmol/L), cognitive dysfunction may occur; when blood glucose decrease to 54 mg/dL (3.0 mmol/L) and sustains for a long time, it may cause brain death [11]. Perioperative hypoglycemia, especially repeated and severe hypoglycemia, may negatively affect the health outcomes of the patients, leading to increased morbidity and mortality, length of stay, readmissions, and health care expenditures [9, 10]. In addition, it may also bring about a huge socio-economic burden to the family and society as a whole [9, 10]. It is thus both important and urgent to assess the risk factors of perioperative hypoglycemia among patients with T2DM, which is beneficial to guide for future effective intervention programs to reduce the risk of perioperative hypoglycemia and its related adverse health outcomes.

The past literature has seen extensive efforts devoted to studying the risk factors of hypoglycemia in patients with T2DM both in China and abroad. A review of these risk factors has produced five major categories: general factors, therapeutic factors, disease-related factors, metabolic index factors, and lifestyle-related factors [12]. General factors mainly include age, duration of diabetes, body mass index (BMI), length of hospital stay, education level, previous history of hypoglycemia, previous experience of hypoglycemia health education, etc. Therapeutic factors mainly include the administration of sulfonylureas hypoglycemic drugs and insulin, etc. Disease-related factors mainly include average blood glucose level, blood glucose variability, diabetic complications, other diseases, surgery, pregnancy, etc. Metabolic index factors mainly include glycosylated hemoglobin (HbA1c), renal function, and triglycerides, etc. Lifestyle-related factors mainly include diet, exercise, sleep, and mood, etc. Among those risk factors, basal insulin dose, the variation coefficient of blood glucose (BG), and previous hypoglycemic episodes were identified as the three strongest predictors of iatrogenic hypoglycemia in hospitalized patient [13].

However, few studies have reported the risk factors or developed a risk prediction model of perioperative hypoglycemia in hospitalized T2DM patients undergoing elective surgery. The exploration of risk factors and the construction of a simple risk prediction model may help to timely detect, intervene and prevent the occurrence of perioperative hypoglycemia. Therefore, our research was conducted to develop a predictive model to assess the perioperative hypoglycemia risk in hospitalized T2DM patients undergoing elective surgery. Our findings may provide useful guidance to support clinicians in predicting perioperative hypoglycemia risk, so as to initiate early intervention.

## Methods

### Patient identification and eligibility

Our study retrospectively analyzed the electronic medical records of T2DM patients who underwent elective surgery at Xiangya Hospital of Central South University in Southern China from July 2019 to December 2019. Inclusion criteria included: (1) patients with a diagnosis of T2DM [12] who underwent elective surgery [including general surgery, cardiothoracic surgery, neurosurgery, gynecological surgery, burn plastic surgery, interventional surgery, orthopedic surgery, urological surgery, ENT (ear–nose–throat) surgery]; (2) aged 18 to 80 years; (3) with available full information on T2DM related clinical indicators. Exclusion criteria included: (1) patients with type 1 diabetes, gestational diabetes, or other special types of diabetes; (2) patients with emergency surgery, two or more surgeries, or cancellation of surgery; (3) patients without blood glucose monitoring results during hospitalization or patients received insulin injections in the operating room; (4) patients with more than 40% of medical records data missing.

This study complied with the principles of the Declaration of Helsinki and was approved by the Ethics Committee of Xiangya Hospital of Central South University (Approval No. 2019121178). The case information of all participants was collected anonymously with the consent of hospital management. All procedures were performed in accordance with institutional guidelines.

### Predictor variables and outcome measures

Through the network information center, the homepage of electronic medical records from July to December 2019 was searched using the keywords “type 2 diabetes” and “elective surgery”, and all patient information that met the two conditions was screened. After unified training, the survey team used self-designed questionnaires to collect patient general information by consulting the patient’s medical records. The predictor variables included general factors (gender, age, duration of diabetes, BMI, education level, place of residence, and family history), therapeutic factors (fasting before surgery, preoperative hypoglycemic regimen, and surgery day hypoglycemic plan), disease-related factors (glycemic variability [GV], hypertension history, type of anesthesia, and surgery grade), and metabolic index factors (glycosylated hemoglobin [HbA1c] and urine sugar). GV was quantified using the standard deviation of blood glucose (SDBG), which is defined as the standard deviation of rapid blood glucose or venous blood glucose value during hospitalization [14]. The surgical grade was based on the difficulty, complexity and risk of surgery in the 2018 Ministry of Health’s surgical-grade classification catalog in China. For T2DM patients whose blood sugar were controlled in the basis of the suggested target range (6.7–11.1 mmol/L) [15], intraoperative blood glucose was monitored every 2 h. For T2DM patients who received subcutaneous or intravenous insulin therapy during the operation or whose blood glucose levels were not within the target range, blood glucose was monitored every hour [15]. Rapid arterial blood glucose monitoring was routinely performed during operation. The brain state monitoring was used routinely in patients under general anesthesia and the main monitoring index was the bispectral index (BIS). Anesthesiologists are mainly responsible for the management of blood sugar in patients under general anesthesia, and they dynamically adjust the dosage of insulin and glucose to ensure that blood sugar is controlled in the target range during operation. The glucose solutions were routinely given in perioperative volume deficiency compensation. For patients with other anesthesia methods, blood sugar management is the same as no-diabetes patients.

The outcome variable was the risk of hypoglycemia among T2DM inpatients during the perioperative period, which is a binary variable including Hypo^<4.0^ and Hypo^<3.0^. Hypoglycemia was determined using random blood glucose monitoring with the qualified glucometer. Hypo^<4.0^ (Level 1) hypoglycemia was defined as a measurable glucose concentration of between 54 mg/dL (3.0 mmol/L) to 70 mg/dL (3.9 mmol/L). Hypo^<3.0^ (Level 2) hypoglycemia was defined as a blood glucose concentration of < 54 mg/dL (3.0 mmol/L), which was the threshold of neuroglycopenic symptoms that required immediate action to resolve the hypoglycemic event [16].

### Statistical analysis

Statistical analyses were performed using SPSS 26.0 (IBM, USA). Normally distributed continuous data were described by means and standard deviations. Independent t-tests were used for two-group comparisons. Categorical data were described by frequencies, proportions, or percentages. Pearson’s Chi-square tests were used for intergroup comparisons. A backward stepwise logistic regression model was constructed, with hypoglycemia as the dependent variable and all potential predictor factors mentioned above as independent variables to screen for perioperative hypoglycemia risk factors. The receiver operating characteristic (ROC) curve was used to assess model accuracy, with a recommended level of > 0.70 indicating good discrimination to distinguish between subjects who do vs do not develop the outcome. The bootstrap method was used for internal verification. The Hosmer–Lemeshow test was used to verify the calibration degree of the established logistic regression model, i.e., the extent to which the predicted probability of hypoglycemia over or underestimates the observed risk of perioperative hypoglycemia.

## Results

### Univariate analysis of risk factors for hypoglycemia

A total of 1410 patients with T2DM inpatients who underwent elective surgery between July 2019 and December 2019 were included in our analysis. There were 804 males (57%), and the average age was 62 years (SD: 11). The incidence of level 1 and level 2 hypoglycemia were 10.7% and 1.8%, respectively. Table 1 shows the comparisons of risk factors between the hypoglycemia group and the non-hypoglycemia group by univariate analysis. Compared to the non-hypoglycemia group, the hypoglycemia group were more likely to: have an illness duration of ≥ 10 years (61.6% vs 36.1%, P < 0.001), have BMI lower than 24 (68.9% vs 46.2%, P < 0.001), have hospital stay of ≥ 20 days (26.5% vs 7.5%, P < 0.001), live in rural areas (58.9% vs 49.1%, P = 0.032), have fasting before surgery (54.9% vs 38.7%, P < 0.001), have subcutaneous injection of insulin both as preoperative hypoglycemic regimen (47.0% vs 21.5%, P < 0.001) and as surgery day hypoglycemic plan (31.1% vs 13.2%, P < 0.001), have SDBG ≥ 3 mmol/L (47.7% vs 31.0%, P < 0.001), have third level surgical degree (46.4% vs 22.4%, P < 0.001), and have general anesthesia (41.7% vs 33.0%, P = 0.024).

**Table 1:** Comparisons of risk factors between hypoglycemia group and non-hypoglycemia group by univariate analysis (n = 1410)

Variable	Hypoglycemia group (n = 151)	Non-hypoglycemia group (n = 1259)	χ^2^	P value
Male n (%)	80 (53.0)	724 (57.5)	1.104	0.293
Age > 70 years n (%)	46 (30.5)	275 (21.8)	5.699	0.017
Duration of diabetes ≥ 10 years n (%)	93 (61.6)	454 (36.1)	32.136	< 0.001
BMI (kg/m^2^) n (%)			21.518	< 0.001
< 18.5	10 (6.6)	23 (1.8)		
18.5–23.9	94 (62.3)	521 (41.4)		
≥ 24	47 (31.1)	715 (56.8)		
Length of hospital stay (day)			81.145	< 0.001
< 10	49 (32.4)	821 (65.2)		
10~	62 (41.1)	344 (27.3)		
≥ 20	40 (26.5)	94 (7.5)		
Education > 9 years n (%)	98 (64.9)	763 (60.6)	1.285	0.257
Place of residence n (%)			4.579	0.032
Rural	89 (58.9)	618 (49.1)		
Urban	62 (41.1)	641 (50.9)		
Family history of diabetes n (%)	17 (11.3)	147 (11.7)	0.126	0.722
Fasting before surgery n (%)	83 (54.9)	487 (38.7)	14.883	< 0.001
Preoperative hypoglycemic regimen n (%)			47.896	< 0.001
Oral insulin	58 (38.4)	689 (54.7)		
Subcutaneous injection of insulin	71 (47.0)	271 (21.5)		
Others^a^	22 (14.6)	299 (23.8)		
Surgery day hypoglycemic plan n (%)			42.703	< 0.001
Oral insulin	22 (14.6)	407 (32.3)		
Subcutaneous injection of insulin	47 (31.1)	166 (13.2)		
Others^a^	82 (54.3)	686 (54.5)		
SDBG ≥ 3 mmol/L n (%)	72 (47.7)	390 (31.0)	17.233	< 0.001
Hypertension history n (%)	45 (29.8)	459 (36.5)	2.601	0.107
Surgical grade n (%)			47.812	< 0.001
First level	31 (20.5)	540 (42.9)		
Second level	37 (24.5)	316 (25.1)		
Third level	70 (46.4)	282 (22.4)		
Fourth level	13 (8.6)	121 (9.6)		
General anesthesia n (%)	63 (41.7)	415 (33.0)	5.122	0.024
Urine sugar n (%)	44 (29.1)	334 (26.5)	0.871	0.351
HbA1c ≥ 6.5% n (%)	104 (68.9)	904 (71.8)	0.389	0.533

*BMI* body mass index, *HbA1c* glycated hemoglobin, *SDBG* standard deviation of blood glucose

^a^Others include diet, combination treatment, intravenous insulin, and insulin pump, etc.

### Logistic regression analysis of predictors for hypoglycemia

Starting with 16 potential risk factors, the stepwise selection model eliminated 6 variables, thus reducing the list of potential predictors of perioperative hypoglycemia to 10 variables, including age, course of disease, BMI, place of residence, fasting before surgery, general anesthesia, preoperative hypoglycemic regimen, surgery day hypoglycemic plan, SDBG, and surgery grade. Incorporating these variables into the logistic regression equation produced a final model for the predictors of perioperative hypoglycemia, which included duration of diabetes, BMI, SDBG, and preoperative hypoglycemic regimen. Duration of diabetes ≥ 10 year, BMI < 18.5 kg/m^2^, SDBG ≥ 3.0 mmol/L, and subcutaneous injection of insulin as preoperative hypoglycemic regimen were the main risk factors for perioperative hypoglycemia (Table 2).

**Table 2:** Logistic regression analysis the risk factors of perioperative hypoglycemia

Variables	Beta coefficient	SE	Wald	OR	95%CI
Intercept	− 3.462	0.447	59.941		
Duration of diabetes (< 10 years = 0, ≥ 10 years = 1)	1.006	0.303	11.058	2.736	1.512–4.951
BMI (≥ 18.5 kg/m^2^ = 0, < 18.5 kg/m^2^ = 1)	1.636	0.695	5.546	5.133	1.316–20.028
SDBG (< 3 mmol/L = 0, ≥ 3 mmol/L = 1)	1.057	0.290	13.254	2.879	1.629–5.087
Preoperative hypoglycemic regimen (Others^a^ and Oral = 0, Subcutaneous injection of insulin = 1)	0.600	0.296	4.102	1.822	1.020–3.256

*OR* odds ratio, *BMI* body mass index, *SDBG* standard deviation of blood glucose

^a^Others include diet, combination treatment, intravenous insulin, and insulin pump, etc

### Development of a risk prediction model

Based on the logistic regression analysis, a perioperative hypoglycemia risk prediction model for T2DM was constructed as: Logit (P) = − 3.462 + 1.636 * BMI (0. ≥ 18.5 kg/m^2^, 1. < 18.5 kg/m^2^) + 1.006 * duration of diabetes (0. < 10 year, 1. ≥ 10 year) + 1.057 * SDBG (0. < 3.0 mmol/L, 1. ≥ 3.0 mmol/L) + 0.600 * preoperative hypoglycemic regimen (0. oral treatment and others, 1. subcutaneous injection of insulin). The model was statistically significant (likelihood ratio Chi-square = 44.490, degree of freedom = 9, and P < 0.001), and Akaike information criterion (AIC) was 327.585.

### Validation of the risk prediction model

**Fig. 1:**
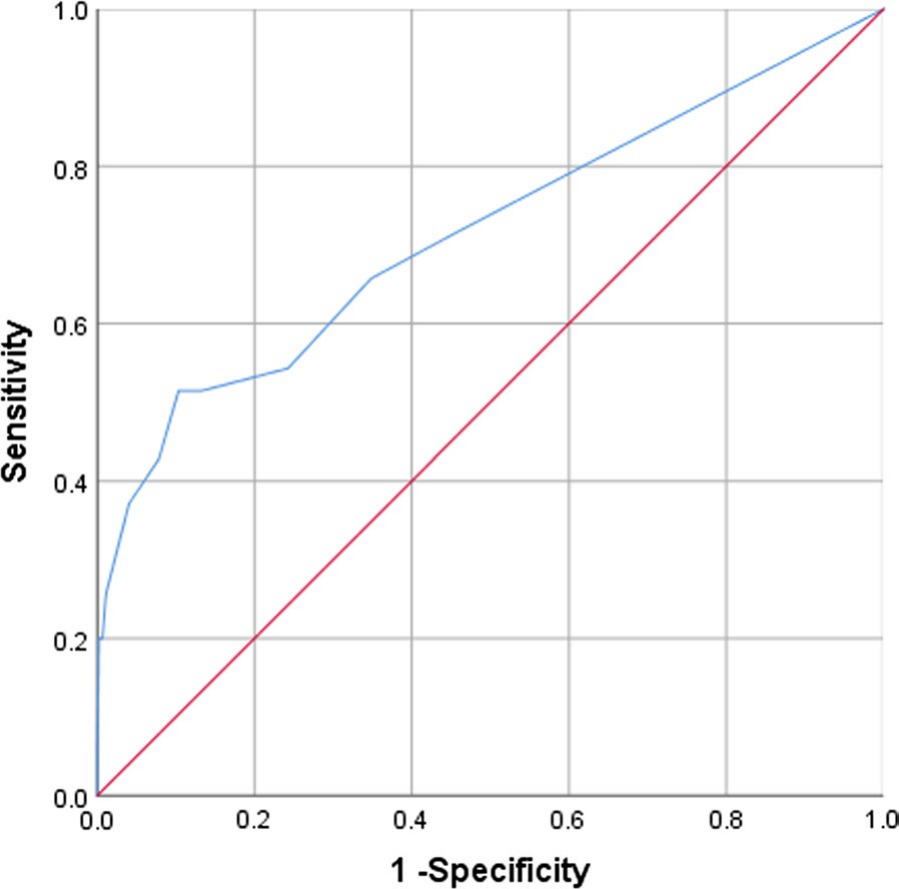
ROC curve of perioperative hypoglycemia probability in hospitalized T2DM patients undergoing elective surgery

Two methods were used to validate the logistic regression model: the ROC curve for model accuracy and the Hosmer–Lemeshow test for calibration degree. The ROC curve showed an area under the curve (AUC) of 0.715 (95% CI 0.610–0.821, P < 0.001) (Fig. 1), indicating the model has good discriminative ability to correctly identify 71.5% of T2DM patients undergoing elective surgery at risk of perioperative hypoglycemia. The Hosmer–Lemeshow test showed no significant differences between predicted and observed risk with a P-value of 0.765 and a Chi-square of 4.926, indicating the model has a good degree of calibration.

### Rule for predicting perioperative hypoglycemia risk

Based on the risk prediction model, a perioperative hypoglycemic risk prediction rule was formed. As shown in Table 3, perioperative hypoglycemia risk was classified into three levels based on the number of positive answers (“yes”) on the four questions: ≥ 3, high risk; 2, medium risk; and ≤ 1, low risk.

**Table 3:** Perioperative hypoglycemia risk assessment form for hospitalized T2DM patients undergoing elective surgery

Items	Yes		No
Duration of diabetes ≥ 10 years	□		□
BMI < 18.5 kg/m^2^	□		□
SDBG ≥ 3 mmol/L	□		□
Preoperative hypoglycemic regimen of insulin subcutaneously	□		□
Perioperative hypoglycemia risk	□ High	□ Medium	□ Low

*BMI* body mass index, *SDBG* standard deviation of blood glucose

## Discussion

We developed a simple and easy prediction tool to identify the risk of perioperative hypoglycemia among hospitalized T2DM patients undergoing elective surgery. The prediction model is mainly composed of four predictors, namely duration of diabetes ≥ 10 years, BMI < 18.5 kg/m^2^, SDBG ≥ 3.0 mmol/L, and subcutaneous injection of insulin as the preoperative hypoglycemic regimen. Internal validation of the prediction model showed high discrimination by ROC curve and good calibration by Hosmer–Lemeshow test.

Our study showed an incidence of 10.7% for level 1 hypoglycemia and 1.8% for level 2 hypoglycemia during the perioperative period. These results were similar to the findings of Chen YF, who reported an incidence of 14% for hypoglycemia in Chinese hospitalized patients with diabetes [17]. However, these results were lower than the reported incidence of 21.5% for level 1 hypoglycemia and 9.6% for level 2 hypoglycemia in Ruan et al.’s study on patients with diabetes admitted to an Oxford University hospital [18]. This discrepancy may be explained by the fact that our study did not include patients with type 1 diabetes. The high incidence of perioperative hypoglycemia poses a major challenge for perioperative management, it is thus important and urgent to pre-assess the risk of perioperative hypoglycemia before surgery and strengthen management to reduce its incidence.

Patients at risk for perioperative hypoglycemia, whether they are diabetic or not, deserve special attention before operation. Currently, there are limited data to suggest that preoperative risk assessment and a prediction model of perioperative hypoglycemia risk in patients with T2DM undergoing surgery have an impact on their outcomes [18, 19]. Given the heterogeneous nature of hospitalized T2DM patients, it is unlikely that one standard of perioperative glycemic control fits all patients. Our research developed and verified a simple perioperative hypoglycemia risk model for T2DM patients undergoing elective surgery, which addresses the need for easy and quick assessment of hypoglycemia risk on busy clinical occasions. This model includes four indicators, duration of diabetes, BMI, SDBG, and subcutaneous injection of insulin as preoperative hypoglycemic regimen. Based on this model, we further categorize patients into three risk levels: low risk, medium risk, and high-risk. This risk prediction model and rule serve as an effective and easy-to-administer assessment tool for clinicians to screen for high-risk patients so as to guide for early intervention. Some measures may include strengthening the frequency of blood glucose monitoring and initiating early intervention for high-risk patients in advance to reduce the risk of perioperative hypoglycemia.

Our study found that a longer duration of diabetes was an independent risk factor for perioperative hypoglycemia. Compared to patients with duration of diabetes < 10 years, patients with duration of diabetes ≥ 10 years had 2.736 times increased risk of hypoglycemia. This finding was consistent with previous studies showing a positive association between the duration of diabetes and hypoglycemia risk. For instance, Gu et al.’s study showed that a longer duration of diabetes was associated with mild hypoglycemia among Chinese patients with T2DM [20]. Dailey et al.’s study also showed that the incidence of nocturnal hypoglycemia was positively correlated with the duration of diabetes among patients receiving NPH insulin therapy [21]. This finding suggests that medical staff should pay special attention to patients with longer diabetes duration during the perioperative period and take effective preventive measures to reduce their risk of hypoglycemia.

Our study found that lower BMI was an independent risk factor for perioperative hypoglycemia. Compared to patients with BMI ≥ 18.5 kg/m^2^, patients with BMI < 18.5 kg/m^2^ had 5.133 times increased risk of perioperative hypoglycemia. This finding was also consistent with previous studies showing that lower BMI was independently associated with increased risk of hypoglycemia and higher mortality [22, 23]. Our findings suggest that special attention should be paid to T2DM patients with lower BMI to reduce their risk of hypoglycemia in the future, which may include strengthening nutritional assessment and initiating nutritional interventions to increase their BMI to prevent hypoglycemia [15].

GV, defined as the degree of glucose level excursion over time, was another independent risk factor for perioperative hypoglycemia. According to previous studies, GV was associated with a range of adverse perioperative outcomes including increased length of stay, higher rates of hypoglycemia, and more perioperative complications [24–26]. As a result, GV has been listed as a predictor of severe hypoglycemia and mortality [24–26]. In this study, we used SDBG to reflect the change of GV and found higher SDBG was associated with an increased risk of perioperative hypoglycemia. Compared to diabetic patients with SDBG < 3.0 mmol/L, patients with SDBG ≥ 3.0 mmol/L had 2.897 times increased risk of perioperative hypoglycemia. This finding was similar to Yuan et al.’s [25] study showing higher perioperative SDBG was associated with increased perioperative GV and higher rates of hypoglycemia in T2DM patients undergoing orthopedic surgery. Our findings suggest that it is particularly important to monitor and manage preoperative blood glucose in patients with T2DM undergoing elective surgery to prevent their risk of perioperative hypoglycemia.

The insulin hypoglycemic program has been recognized as the main risk factor of hypoglycemia, which was also shown in our study. Compared to diabetic patients who used other hypoglycemic regimens, patients who used a subcutaneous injection of insulin as a preoperative hypoglycemic regimen had 1.822 times increased risk of hypoglycemia. This finding was consistent with previous studies showing a positive association between subcutaneous injection of insulin and perioperative hypoglycemia. For instance, Akirov et al.’s study showed that subcutaneous injection of insulin was associated with a fourfold increased risk of hypoglycemia among hospitalized diabetic patients [27]. Kim et al.’s study among a Korean population also showed that only 9% of patients received insulin treatment, about 48% would go to the emergency room due to hypoglycemia [28]. Our findings suggest the necessity of choosing an appropriate preoperative hypoglycemic regimen for patients with elective surgery according to clinical guidelines. For patients with T2DM who use insulin therapy, perioperative and postoperative blood glucose monitoring should be strengthened to assess the risk of hypoglycemia. At the same time, medical staff who intend to use insulin during the perioperative period should receive strengthened special training related to hypoglycemia risk assessment and control [15].

There are several limitations to this study. The sample comes from the same hospital, and the data comes from a retrospective review of the electronic medical record system. The influencing factors included in the analysis may not be comprehensive enough, and other potential factors such as the length of operation, intraoperative medication, intraoperative blood loss, infusion volume and nutritional assessment were not included, which may lead to biased analyses and results. In the future, it is necessary to conduct multi-center studies and use prospective research design to explore a more accurate and comprehensive predicting model.

## Conclusions

We have developed a simple and easy perioperative hypoglycemia risk prediction model among T2DM patients undergoing elective surgery, which contains four predictors: duration of diabetes, BMI, SDBG, and subcutaneous injection of insulin as a preoperative hypoglycemic regimen. Based on this model, patients are further classified into low, medium, and high-risk groups. The model is well-validated with high discrimination and good calibration. The model serves as a favorable tool for clinicians to screen for hypoglycemic risk and guide future interventions to reduce hypoglycemia risk. And multidisciplinary collaboration in research and data collection may also be an effective way to improve the predictive power of models.

## Data Availability

The datasets generated and analysed during the current study are not publicly available due limitation of the affiliation but are available from the corresponding author on reasonable request.
